# Birds Kept in the German Zoo “Tierpark Berlin” Are a Common Source for Polyvalent *Yersinia pseudotuberculosis* Phages

**DOI:** 10.3389/fmicb.2021.634289

**Published:** 2022-01-03

**Authors:** Jens Andre Hammerl, Andrea Barac, Anja Bienert, Aslihan Demir, Niklas Drüke, Claudia Jäckel, Nina Matthies, Jin Woo Jun, Mikael Skurnik, Juliane Ulrich, Stefan Hertwig

**Affiliations:** ^1^Department of Biological Safety, German Federal Institute for Risk Assessment, Berlin, Germany; ^2^Department of Aquaculture, Korea National College of Agriculture and Fisheries, Jeonju, South Korea; ^3^Department of Bacteriology and Immunology, Medicum, Human Microbiome Research Program, Faculty of Medicine, University of Helsinki, Helsinki, Finland; ^4^Division of Clinical Microbiology, HUSLAB, Helsinki University Hospital, Helsinki, Finland

**Keywords:** *Yersinia pseudotuberculosis*, phage, genome, diversity, *Yersinia*, zoo animals

## Abstract

*Yersinia pseudotuberculosis* is an important animal pathogen, particularly for birds, rodents, and monkeys, which is also able to infect humans. Indeed, an increasing number of reports have been published on zoo animals that were killed by this species. One option to treat diseased animals is the application of strictly lytic (virulent) phages. However, thus far relatively few phages infecting *Y. pseudotuberculosis* have been isolated and characterized. To determine the prevalence of *Y. pseudotuberculosis* phages in zoo animals, fecal samples of birds and some primates, maras, and peccaries kept in the Tierpark Berlin were analyzed. Seventeen out of 74 samples taken in 2013 and 2017 contained virulent phages. The isolated phages were analyzed in detail and could be allocated to three groups. The first group is composed of 10 T4-like phages (PYps2T taxon group: *Myoviridae*; *Tevenvirinae*; *Tequatrovirus*), the second group (PYps23T taxon group: *Chaseviridae*; *Carltongylesvirus*; *Escherichia* virus ST32) consists of five phages encoding a podovirus-like RNA polymerase that is related to an uncommon genus of myoviruses (e.g., *Escherichia coli* phage phiEcoM-GJ1), while the third group is comprised of two podoviruses (PYps50T taxon group: *Autographiviridae*; *Studiervirinae*; *Berlinvirus*) which are closely related to T7. The host range of the isolated phages differed significantly. Between 5.5 and 86.7% of 128 *Y. pseudotuberculosis* strains belonging to 20 serotypes were lysed by each phage. All phages were additionally able to lyse *Y. enterocolitica* B4/O:3 strains, when incubated at 37°C. Some phages also infected *Y. pestis* strains and even strains belonging to other genera of *Enterobacteriaceae*. A cocktail containing two of these phages would be able to lyse almost 93% of the tested *Y. pseudotuberculosis* strains. The study indicates that *Y. pseudotuberculosis* phages exhibiting a broad-host range can be isolated quite easily from zoo animals, particularly birds.

## Introduction

The genus *Yersinia* is currently composed of 28 species of which three are known to be pathogenic for humans.^1^ *Yersinia pestis* is the causative agent of Plague. *Yersinia pseudotuberculosis* and *Y. enterocolitica* are enteropathogenic species which may cause diseases termed yersiniosis (Carniel, 2001). Yersiniosis is the fourth most common bacterial enteritis in Europe, which is mainly caused by *Y. enterocolitica* (European Food Safety Authority, 2021). However, an increasing number of human yersiniosis outbreaks have also been reported for *Y. pseudotuberculosis* (Inoue et al., 1984; Tertti et al., 1984; Nakano et al., 1989; Press et al., 2001; Hallanvuo et al., 2003; Jalava et al., 2004, 2006; Nuorti et al., 2004; Rimhanen-Finne et al., 2009; Nakamura et al., 2013; Pärn et al., 2014; Vasala et al., 2014; Williamson et al., 2016). This species is very closely related to *Y. pestis* and therefore, it is not surprising that *Y. pseudotuberculosis* and *Y. enterocolitica* have different reservoirs. In Europe, the presence of *Y. enterocolitica* is clearly associated with pigs (Fredriksson-Ahomaa et al., 2006a,b). Infections by this species are mainly caused by the consumption of contaminated pork (Laukkanen et al., 2008). By contrast, the host spectrum of *Y. pseudotuberculosis* is more diverse, as this species is mainly an animal pathogen, which more rarely infects humans (Nikolova et al., 2001; Nakamura et al., 2015). Indeed, there is a growing number of reports describing the occurrence of *Y. pseudotuberculosis* in, e.g., wild boars and zoo animals (Iwata et al., 2008; Fredriksson-Ahomaa et al., 2011; Fogelson et al., 2015; Nakamura et al., 2015; Arrausi-Subiza et al., 2016; Reinhardt et al., 2018a,b). *Y. pseudotuberculosis* infections and outbreaks among zoo animals have been a problem for decades and therefore, vaccinations are in use in some zoos. Particularly birds quite often carry this pathogen, which can be lethal for these animals (Cork et al., 1999; Galosi et al., 2015; Nakamura et al., 2016; Shopland et al., 2020). Treatment with antibiotics might be an option but multidrug resistance encoded by conjugative plasmids has already been described for *Y. pseudotuberculosis* (Cabanel et al., 2017). To prevent further resistance development, one alternative approach to treat bacterial infections is the application of virulent (strictly lytic) phages. Phages are viruses which exclusively infect bacteria. They generally have a narrow host range and occur everywhere where their hosts are living. Moreover, virulent phages are also effective against multidrug-resistant bacteria and have a self-replicative mode of action (Wang et al., 2010). On the other hand, temperate phages may integrate their genome into the bacterial chromosome or may reside as a plasmid, which is then called a prophage. Since temperate phages sometimes contain virulence or antibiotic resistance genes, which can be transmitted by horizontal gene transfer to other bacteria, they are not suited for phage therapy (Owens et al., 2013). Compared to the two other human-pathogenic *Yersinia* species, there are only few reports on the isolation and characterization of *Y. pseudotuberculosis* phages, although some *Y. pestis* phages are able to lyse *Y. pseudotuberculosis* and even showed a lytic activity on certain *Escherichia coli*, *Klebsiella pneumoniae*, and *Shigella sonnei* strains (Filippov et al., 2012; Rashid et al., 2012). The broad-host range phages PY100, vB_YenP_Rambo (Rambo) and vB_YenM_P281 (P281) did not only infect the three pathogenic, but also some non-pathogenic *Yersinia* species (Schwudke et al., 2008; Hammerl et al., 2021). By contrast, eight phages exclusively infecting *Y. pseudotuberculosis* O:1a strains were isolated from pig stools (Salem et al., 2015). All stools from which these phages were recovered, were negative for *Y. pseudotuberculosis* O:1a suggesting that the phages might have replicated in another host (Salem et al., 2015). Recently, three other T4-like phages from pig stools infecting *Y. pseudotuberculosis* and *Y. pestis* using LPS and OmpF as receptors were reported (Salem et al., 2021).

In this work for the first-time, fecal samples from animals kept in a zoo in Berlin were screened for the presence of phages able to infect *Y. pseudotuberculosis*. We wanted to know whether zoo animals may carry virulent or temperate *Y. pseudotuberculosis* phages and which properties they have. The isolated phages were characterized in terms of their morphology, host range, and genome composition with special emphasis on tail fiber proteins that are important determinants for host specificity.

## Materials and Methods

### Bacterial Strains and Culture Conditions

Overall, 287 bacterial strains used in this study were selected from the strain collections of the Consiliary Laboratory for *Yersinia* at the German Federal Institute for Risk Assessment (BfR), Berlin, Germany and of the Skurnik laboratory at University of Helsinki, Finland. Information on the species and serotypes of strains is given in Tables 1 and 2. If not otherwise indicated, *Yersinia* sp. bacteria were cultivated in lysogeny broth (LB) at 28°C with continuous shaking at 200–225 rpm (Hammerl et al., 2008), while other *Enterobacteriaceae* were incubated in LB at 37°C.

**Table 1:** Host range of the phages on *Yersinia*.

Phage group	PYps2T										PYps23T					PYps50T	
	PYps2T	PYps5T	PYps10T	PYps11T	PYps14T	PYps15T	PYps32T	PYps35T	PYps47T	PYps55T	PYps3T	PYps4T	PYps16T	PYps16N	PYps23T	PYps49T	PYps50T
***Y. pseudotuberculosis* strains**																	
**(*n* = 128)**	**25 (19.5%)**	**19 (14.8%)**	**58 (45.3%)**	**45 (35.2%)**	**78 (60.9%)**	**85 (66.4%)**	**87 (68.0%)**	**106 (82.8%)**	**52 (40.6%)**	**79 (61.7%)**	**8 (6.3%)**	**10 (7.8%)**	**7 (5.5%)**	**8 (6.3%)**	**11 (8.6%)**	**111 (86.7%)**	**111 (86.7%)**
O:1a (*n* = 27)	6	8	25	17	27	26	27	26	21	24	–	–	–	–	–	27	27
O:1b (*n* = 16)	5	2	10	10	8	11	12	15	5	10	1	1	1	1	1	14	14
O:1^*^ (*n* = 4)	2	–	2	2	2	2	2	3	1	3	–	–	–	–	–	3	3
O:2a (*n* = 7)	4	7	6	6	7	7	7	7	5	7	–	–	–	–	–	6	5
O:2b (*n* = 3)	–	1	1	1	2	2	2	3	1	2	1	1	1	1	1	1	2
O:2c (*n* = 3)	–	1	1	1	3	3	3	3	1	3	–	1	–	1	1	2	2
O:3 (*n* = 19)	1	–	1	–	14	15	14	17	10	15	–	–	–	–	–	17	18
O:4a (*n* = 2)	1	–	2	2	1	1	1	2	–	–	2	2	1	2	2	2	2
O:4b (*n* = 3)	2	–	–	–	2	2	2	3	2	3	–	–	–	–	–	2	2
O:4^*^ (*n* = 3)	–	–	–	–	–	–	–	–	–	–	3	3	3	3	3	2	3
O:5a (*n* = 5)	–	–	–	–	–	–	–	2	–	–	–	–	–	–	–	4	4
O:5b (*n* = 3)	1	–	1	–	1	2	1	2	–	2	–	–	–	–	–	2	2
O:6 (*n* = 3)	–	–	–	–	1	2	3	1	1	–	–	1	–	–	1	2	1
O:7 (*n* = 4)	–	–	1	–	1	1	1	3	1	–	–	–	–	–	–	4	4
O:8 (*n* = 2)	–	–	1	–	1	1	1	1	1	1	–	–	–	–	–	2	2
O:9 (*n* = 4)	–	–	–	–	–	–	2	–	–	–	–	–	–	–	2	4	2
O:10 (*n* = 3)	–	–	–	–	–	1	–	3	–	1	–	–	–	–	–	2	2
O:11 (*n* = 2)	–	–	–	–	–	–	–	1	–	–	1	1	1	–	–	1	1
O:12 (*n* = 2)	1	–	1	1	–	–	–	2	–	2	–	–	–	–	–	2	2
O:13 (*n* = 2)	1	–	1	1	1	2	1	2	–	1	–	–	–	–	–	2	2
O:14 (*n* = 1)	1	–	–	–	1	1	1	1	–	1	–	–	–	–	–	1	1
O:15 (*n* = 2)	–	–	–	–	–	–	–	2	–	1	–	–	–	–	–	1	2
NT (*n* = 9)	–	–	5	4	6	6	7	7	3	3						8	8
***Y. pestis* strains**																	
**(*n* = 9) rough**	**2 (22.2%)**	**0 (0.0%)**	**0 (0.0%)**	**1 (11.1%)**	**9 (100%)**	**8 (88.9%)**	**8 (88.9%)**	**9 (100%)**	**4 (44.4%)**	**6 (66.7%)**	**0 (0.0%)**	**0 (0.0%)**	**0 (0.0%)**	**0 (0.0%)**	**0 (0.0%)**	**9 (100%)**	**8 (88.9%)**
***Y. wautersii* strains**																	
**(*n* = 2)**	**0 (0%)**	**0 (0%)**	**0 (0%)**	**0 (0%)**	**0 (0%)**	**0 (0%)**	**0 (0%)**	**1 (50%)**	**0 (0%)**	**0 (0%)**	**1 (50%)**	**1 (50%)**	**1 (50%)**	**1 (50%)**	**1 (50%)**	**1 (50%)**	**1 (50%)**
O:4a (*n* = 1)	–	–	–	–	–	–	–	1	–	–	1	1	1	1	1	1	1
O:15 (*n* = 1)	–	–	–	–	–	–	–	–	–	–	–	–	–	–	–	–	–
***Y. similis* strains**																	
**(*n* = 5)**	**1 (20%)**	**1 (20%)**	**1 (20%)**	**1 (20%)**	**0 (0%)**	**0 (0%)**	**0 (0%)**	**5 (100%)**	**0 (0%)**	**1 (20%)**	**1 (20%)**	**1 (20%)**	**1 (20%)**	**1 (20%)**	**1 (20%)**	**5 (100%)**	**5 (100%)**
O:1c (*n* = 1)	–	–	–	–	–	–	–	1	–	–	–	–	–	–	–	1	1
O:6 (*n* = 1)	–	–	–	–	–	–	–	1	–	–	–	–	–	–	–	1	1
O:9 (*n* = 1)	–	–	–	–	–	–	–	1	–	–	–	–	–	–	–	1	1
O:11 (*n* = 1)	–	–	–	–	–	–	–	1	–	–	1	1	1	1	1	1	1
O:12 (*n* = 1)	1	1	1	1	–	–	–	1	–	1	–	–	–	–	–	1	1

* The subtypes of these strains were not determined.

**Table 2:** Lysis of other *Enterobacteriaceae.*

**Phage group**	**PYps2T**										**PYps23T**					**PYps50T**	
	PYps2T	PYps5T	PYps10T	PYps11T	PYps14T	PYps15T	PYps32T	PYps35T	PYps47T	PYps55T	PYps3T	PYps4T	PYps16T	PYps16N	PYps23T	PYps49T	PYps50T
*Citrobacter freundii* (*n* = 3)	–	–	–	–	–	–	–	–	–	–	–	–	–	–	–	–	–
*Enterobacter cloacae* (*n* = 8)	–	–	–	–	–	–	–	–	–	–	–	–	–	–	–	–	–
*Escherichia coli* untyped (*n* = 4)	1	1	1	1	–	–	–	1	–	–	1	1	1	1	1	–	1
*Escherichia coli* O1:K1 (*n* = 1)	1	–	–	–	–	–	–	1	–	–	–	1	–	–	–	1	1
*Escherichia coli* O157:H7 (*n* = 12)	1	1	2	–	–	1	–	1	1	1	1	–	2	2	1	3	5
*Escherichia coli* O104:H4 (*n* = 8)	1	2	2	1	–	–	–	–	–	–	3	1	2	3	4	3	4
*Escherichia coli* O26 (*n* = 6)	–	1	–	–	1	1	1	1	–	1	2	1	1	1	2	1	–
*Escherichia coli* O103 (*n* = 7)	1	–	–	–	1	–	1	–	1	1	1	1	–	2	1	1	1
*Escherichia coli* O88 (*n* = 6)	1	1	–	1	1	–	–	1	–	–	1	–	1	–	–	–	1
*Klebsiella pneumoniae* (*n* = 19)	–	–	–	–	–	–	–	–	–	–	–	–	–	–	–	–	–
*Klebsiella oxytoca* (*n* = 6)	–	–	–	–	–	–	–	–	–	–	–	–	–	–	–	–	–
*Klebsiella michiganensis* (*n* = 4)	–	–	–	–	–	–	–	–	–	–	–	–	–	–	–	–	–
*Klebsiella variicola* (*n* = 3)	–	–	–	–	–	–	–	–	–	–	–	–	–	–	–	–	–
*Klebsiella aerogenes* (*n* = 2)	–	–	–	–	–	–	–	–	–	–	–	–	–	–	–	–	–
*Morganella morganii* (*n* = 37)	–	–	–	–	–	–	–	–	–	–	–	–	–	–	–	1	1
*Proteus mirabilis* (*n* = 5)	–	–	–	–	–	–	–	–	–	–	–	–	–	–	–	–	–
*Providencia rettgeri* (*n* = 6)	–	–	–	–	–	–	–	–	–	–	–	–	–	–	–	–	–
*Salmonella enterica* (*n* = 3)	–	–	–	–	–	–	–	–	–	–	1	1	1	–	–	2	1
*Shigella flexneri* (*n* = 3)	1	–	–	–	–	–	–	–	–	–	–	–	–	–	–	2	2

### Isolation, Propagation, and Purification of Phages

In total, 74 fecal samples taken from 56 birds and 18 mammals (Chacoan peccaries, Patagonian maras, and various monkeys) in the zoo Tierpark Berlin were analyzed in August 2013 and July 2017 (Supplementary Table S1). Samples collected from the ground were immediately suspended on site in approximately 5 ml 0.85% NaCl solution. After arrival in the laboratory, 45 ml of SM buffer (Jäckel et al., 2015) was added to further suspend the samples. Following this, 10 ml of the material was transferred to a centrifuge tube and spun for 20 min at 8,000 rpm and 10°C. The supernatants were passed through 0.45 μm pore-size filters (VWR International, Darmstadt, Germany) and stored at 4°C until they were used to determine lytic activity. This was done by spotting 10 μl of serial dilutions of each sample onto a lawn of 20 *Y. pseudotuberculosis* indicator strains belonging to the serotypes O:1a, b; O:2a, b, c; O:3, O:4a, b, c; O:5a, b; and O:6, O:7. After incubation overnight at 28°C, plaques were inspected. Individual phages were isolated by 3-fold recovery of single plaques. High-titer lysates of the phages were obtained by infecting 200 ml cultures of the indicator strain (OD_588_ = 0.5) with phages at a MOI of 0.01 to 0.1. After cell lysis, lysates were centrifuged for 20 min at 10,000 x g to remove debris and then filtered (see above). Phages were concentrated by ultracentrifugation and purified using CsCl step gradients (Jäckel et al., 2017).

### Host Range Determination

The host range of the phages was determined twice by activity assays. 100 μl of the respective indicator strain was mixed with 6 ml prewarmed NZCYM soft agar (0.6%) and poured onto a LB agar plate (Jäckel et al., 2015). Ten μl of serial dilutions of each lysate (adjusted to ~1 × 10^7^ pfu/ml) was spotted onto the overlay agar. Plates were incubated overnight at 28°C or 37°C. Lytic activity of the phages was identified by single plaques.

### Transmission Electron Microscopy

CsCl-purified phages were investigated by TEM using the negative staining procedure with uranyl acetate. Briefly, drops of phage preparations were applied to pioloform-carbon-coated, 400-mesh copper grids (Plano GmbH, Wetzlar, Germany), incubated for 10 min, and fixed with 2.5% aqueous glutaraldehyde (Taap Laboratories, Aldermaston, United Kingdom) for 1 min. Thereafter, phages were stained with a 2% aqueous uranyl acetate (Merck, Darmstadt, Germany) solution (pH 7.0) for 3 min. Specimens were examined by TEM using a JEM-1010 (JEOL, Tokyo, Japan) at 80 kV acceleration voltage.

### Phage DNA Preparation, Sequencing, and Genome Annotation

For short-read, paired end whole-genome sequencing, phage DNA was extracted from concentrated virions by proteinase K/SDS treatment at 56°C for 2 h as previously described (Jäckel et al., 2015). The phage DNA was used for the preparation of DNA sequencing libraries using the Illumina DNA Flex Library Preparation kit (Illumina, CA, United States). MiSeq sequencing was conducted as previously described (Osieka et al., 2017). Raw reads were subjected to *de novo* assembly using the spades algorithm of the PATRIC database (version 3.6.2). For initial prediction of putative coding sequences, the annotation tool of the PATRIC database was used. Further bioinformatics analysis was conducted using the blast-suite^2^ (BLASTn, BLASTx BLASTp) of the National Center for Biotechnology Information (NCBI). Prediction of potential transcription terminators was conducted using ARNold^3^ (Hofacker et al., 1994; Macke et al., 2001).

Phylogenetic analyses were performed using the CSI Phylogeny tool (version 1.4; default parameters),^4^ of the Center for Genomic Epidemiology (Kaas et al., 2014). The resulting Newick files were further processed with FigTree (version 1.4.4).^5^ Dot Plot analyses were conducted using Accelrys DS Gene (version 2.5; Accelrys Inc.) with in text specified parameters.

### Nucleotide Sequence Accession

The genomes of the *Y. pseudotuberculosis* phages described in this study were deposited in GenBank under the specified accession numbers: PYps2T (MT828551), PYps3T (MW147599), PYps4T (MW147600), PYps5T (MT828552), PYps10T (MT828553), PYps11T (MT515751), PYps14T (MT526905), PYps15T (MT515752), PYps16N (MW147601), PYps16T (MW147602), PYps23T (MW147598), PYps32T (MT515753), PYps35T (MT515754), PYps47T (MT515755), PYps49T (MW147603), PYps50T (MT515757), and PYps55T (MT515756).

## Results

### Isolation, Host Range, and Morphology of 17 *Y. pseudotuberculosis* Phages

The phages described in this study were isolated from fecal samples of animals kept in the Tierpark Friedrichsfelde, Berlin (Germany). In 2013, only samples of birds were investigated, whereas in 2017, some samples of other animals were additionally examined (Supplementary Table S1). After purification of the samples (see “Materials and Methods”), preparations were spotted onto 20 *Y. pseudotuberculosis* strains belonging to various serotypes (e.g., O:1a, O:2a, O:3, O:4a, O:5a, O:6, and O:7). In total, seven (out of 20) and 10 (out of 54) samples collected in 2013 and 2017, respectively, showed lytic activity on at least one indicator strain. With the exception of one phage (PYps23T) isolated from a capuchin monkey (*Cebus*), all positive samples had been taken from birds (Table 3). By contrast, altogether 57 samples did not show lytic activity on any tested indicator strain.

**Table 3:** Origin of the phages analyzed in this study.

Phage ID	Morphology (taxonomic classification)	Lysed species	Source of the fecal sample	Sample no.^*^	Year of sampling
PYps2T	Myovirus (Tequatrovirus)	*Y. pseudotuberculosis*, *Y. pestis*, *Y. similis*, *E. coli*, *S. flexneri*	Rufous hawk owl (*Ninox rufa*)	2013–02	2013
PYps3T	Myovirus (Carltongylesvirus)	*Y. pseudotuberculosis*, *Y. similis*, *Y. wautersii*, *E. coli*, *S. enterica*	Turkmenian eagle-owl (*Bubo bubo omissus*)	2013–03	2013
PYps4T	Myovirus (Carltongylesvirus)	*Y. pseudotuberculosis*, *Y. similis*, *Y. wautersii*, *E. coli*, *S. enterica*	Black-headed ibis (*Threskiornis melanocephalus*)	2013–04	2013
PYps5T	Myovirus (Tequatrovirus)	*Y. pseudotuberculosis*, *Y. similis*, *E. coli*	Turkey vulture (*Cathartes aura*)	2013–05	2013
PYps10T	Myovirus (Tequatrovirus)	*Y. pseudotuberculosis*, *Y. similis*, *E. coli*	Morepork (*Ninox novaeseelandiae*)	2013–10	2013
PYps11T	Myovirus (Tequatrovirus)	*Y. pseudotuberculosis*, *Y. similis*, *Y. pestis*, *E. coli*	White-faced scops owl (*Ptilopsis granti*)	2013–11	2013
PYps16T	Myovirus (Carltongylesvirus)	*Y. pseudotuberculosis*, *Y. similis*, *Y. wautersii*, *E. coli*, *S. enterica*	Snow owl (*Bubo scandiacus*)	2013–16	2013
PYps14T	Myovirus (Tequatrovirus)	*Y. pseudotuberculosis*, *Y. pestis*, *E. coli*	Mixed aviary	2017–14	2017
PYps15T	Myovirus (Tequatrovirus)	*Y. pseudotuberculosis*, *Y. pestis*, *E. coli*	Mixed aviary	2017–15	2017
PYps16N	Myovirus (Carltongylesvirus)	*Y. pseudotuberculosis*, *Y. similis*, *Y. wautersii*, *E. coli*	Mixed aviary	2017–16	2017
PYps23T	Myovirus (Carltongylesvirus)	*Y. pseudotuberculosis*, *Y. similis*, *Y. wautersii*, *E. coli*	Cebus capuchin monkey (*Cebus capucinus*)	2017–23	2017
PYps32T	Myovirus (Tequatrovirus)	*Y. pseudotuberculosis*, *Y. pestis*, *E. coli*	Kennicotti owl/Western screech owl (*Megascops kennicottii*)	2017–32	2017
PYps35T	Myovirus (Tequatrovirus)	*Y. pseudotuberculosis*, *Y. pestis*, *Y. similis*, *Y. wautersii*, *E. coli*	Great horned owl (*Bubo virginianus*)	2017–35	2017
PYps47T	Myovirus (Tequatrovirus)	*Y. pseudotuberculosis*, *Y. pestis*, *E. coli*	Mixed aviary	2017–47	2017
PYps49T	Podovirus (Berlinvirus)	*Y. pseudotuberculosis*, *Y. similis*, *Y. wautersii*, *E. coli*, *M. morganii*, *S. enterica*, *S. flexneri*	Mixed aviary	2017–49	2017
PYps50T	Podovirus (Berlinvirus)	*Y. pseudotuberculosis*, *Y. similis*, *Y. wautersii*, *E. coli*, *M. morganii*, *S. enterica*, *S. flexneri*	Mixed aviary	2017–50	2017
PYps55T	Myovirus (Tequatrovirus)	*Y. pseudotuberculosis*, *Y. pestis*, *Y. similis*, *E. coli*	Mixed aviary	2017–55	2017

* Further information on all investigated samples is given in Supplementary Table S1.

**Figure 1:**
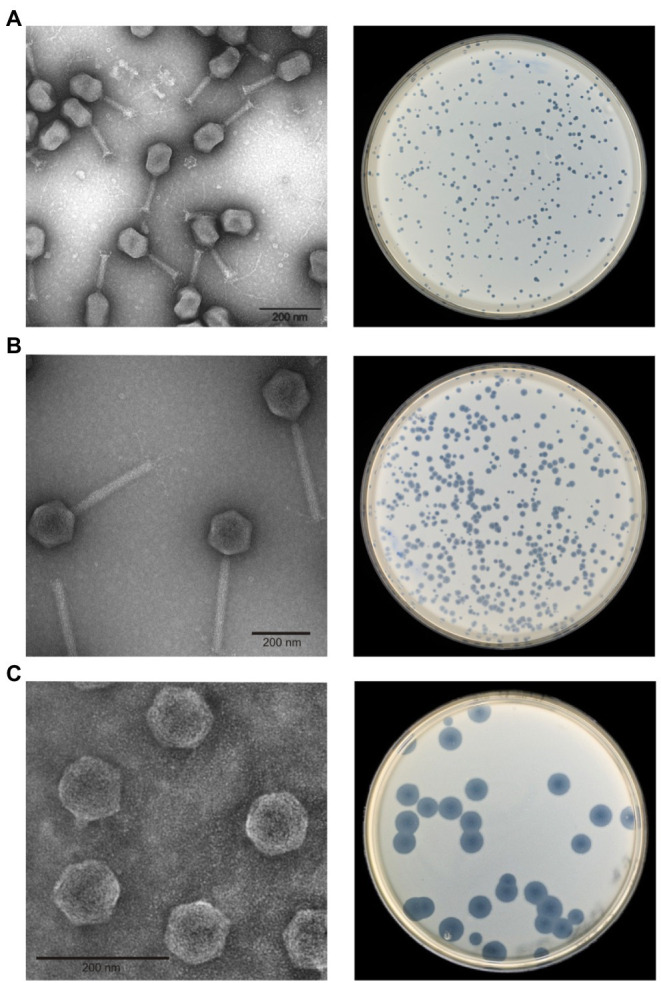
Morphology and plaque formation of *Yersinia pseudotuberculosis* phages. **(A)** PYps2T group, **(B)** PYps23T group, and **(C)** PYps50T group.

After isolation of single plaques, phages were propagated, purified, and analyzed by electron microscopy. Fifteen phages are myoviruses. However, while 10 phages (PYps2T, 5T, 10T, 11T, 14T, 15T, 32T, 35T, 47T, and 55T) exhibited a prolate head (110 × 90 nm) and a rather short tail (length: ~100 nm), the head of five phages (PYps3T, 4T, 16T, 16N and 23T) was isometric (diameter: ~105 nm), and their tail significantly longer (length: ~200 nm, Figure 1). Contracted tails and tail fibers were discernible in most preparations. Unlike the aforementioned phages, two phages (PYps49T and 50T) are podoviruses (Figure 1). They showed a small isometric head (diameter: ~58 nm) and a very short tail. Thus, on the basis of morphology, the investigated phages could be allocated to three groups, the type phages of which are PYps2T, PYps23T, and PYps50T.

The host ranges of the phages were determined by testing 128 *Y. pseudotuberculosis* strains belonging to 20 serotypes and strains belonging to other species. The study showed that all phages lysed several *Y. pseudotuberculosis* serotypes but that the host specificity diverged in part significantly. Between 5.5 and 86.7% of the tested strains were infected by each phage (Table 1). The five myoviruses possessing an isometric head and a long tail (PYps23T group) lysed only few strains which, however, belonged to various serotypes. Most of the remaining myoviruses (PYps2T group) infected many more *Y. pseudotuberculosis* strains. All tested serotypes were lysed by at least one phage. Among the myoviruses, PYps35T exhibited the broadest host range, as it lysed 106 out of 128 (82.8%) strains. More strains (86.7%) were only lysed by the two podoviruses PYps49T and 50T. Plaques produced by the phages revealed different morphologies ranging from very small (diameter less than 1 mm) to very large (approximately 1 cm in size) produced by the podoviruses (Figure 1).

Strains of *Y. pestis*, *Y. similis*, and *Y. wautersii*, which are closely related to *Y*. *pseudotuberculosis*, revealed susceptibility as well. All nine tested *Y. pestis* strains were lysed by the myoviruses PYps14T and PYps35T and the podovirus PYps49T. In addition, the myovirus PYps35T and the podoviruses PYps49T and PYps50T lysed all five *Y. similis* strains and one of two *Y. wautersii* strains belonging to serotype O:4a. Interestingly, this strain was susceptible also to the PYps3T group. As phages of this group were able to lyse several O:4a strains of *Y. pseudotuberculosis*, they might have a preference for this serotype (Table 1). We additionally tested *Y. enterocolitica* strains belonging to the bio/serotypes B4/O:3 (Pärn et al., 2014), B2/0:9 (Press et al., 2001), B2/O:5,27 (Pärn et al., 2014), and 1B:O:8 (Nakano et al., 1989), which cause most human infections in Europe. Surprisingly, all B4/O:3 strains but no strains of the other bio/serotypes were susceptible to the phages. Though, some phages of the PYps2T and PYps23T group only lysed B4/O:3 strains at a temperature of 37°C while the remaining phages were lytic at both temperatures, 37°C and 28°C (data not shown).

It has previously been reported that some *Y. pseudotuberculosis* phages infected species outside the genus *Yersinia*. We therefore examined a number of other enterobacteria. As shown in Table 2, some *E. coli* (16/44), *Morganella morganii* (1/37), *Shigella* (2/3), and *Salmonella* (2/3) strains were lysed by several phages, whereas the species *Citrobacter freundii* (*n* = 3), *Enterobacter cloacae* (*n* = 8), *Klebsiella* spp. (*n* = 34), *Proteus mirabilis* (*n* = 5), and *Providencia rettgeri* (*n* = 6) were not susceptible.

### Members of Each Group Possess Highly Conserved Genomes but Are Not Related to Phages of Other Groups

To study the 17 phages in detail, they were subjected to nucleic acid isolation followed by whole-genome sequencing (see “Materials and Methods”). Sequence analyses indicated that phages within each group are very similar (91.3 to 99.9% identity over 92 to 100% of the genomes), whereas no similarities were detected between the groups. Members of the PYps2T group contain large genomes of approximately 166 to 170 kb and are related to T4-like phages. The strongest similarities exist to the *Escherichia* phages YUEEL01 and vB_EcoM_JB75 (Acc. Nos. KY290975.2 and MH355584.1) with an identity of 99% over 94 and 93% of the covered genome, respectively. PYps23T group members contain smaller genomes (*ca*. 53 to 56 kb). The closest relatives are *Escherichia* phage ST32 (Liu et al., 2018) and Enterobacteria phage phiEcoM-GJ1 (Jamalludeen et al., 2008), to which PYps23T is approximately 92% identical over 70% of the covered genomes. The podoviruses of the PYps50T group have a size of 39.6 kb and are very similar to *Salmonella* phage BSP161 (Acc. no. MG471392.1), to the Enterobacteria phages 285P (Xu et al., 2014) and BA14 (Mertens and Hausmann, 1982) and to *Escherichia* phage vB_EcoP_S523 (Acc. no. MH031343.1), which all are T7-related phages. They exhibit nucleotide identity values of more than 90% over 94% of the covered genomes.

### The PYps2T Group Is Closely Related to T4-like Phages of the Taxid: *Caudovirales*; *Myoviridae*; *Tevenvirinae*; *Tequatrovirus*

**Figure 2:**
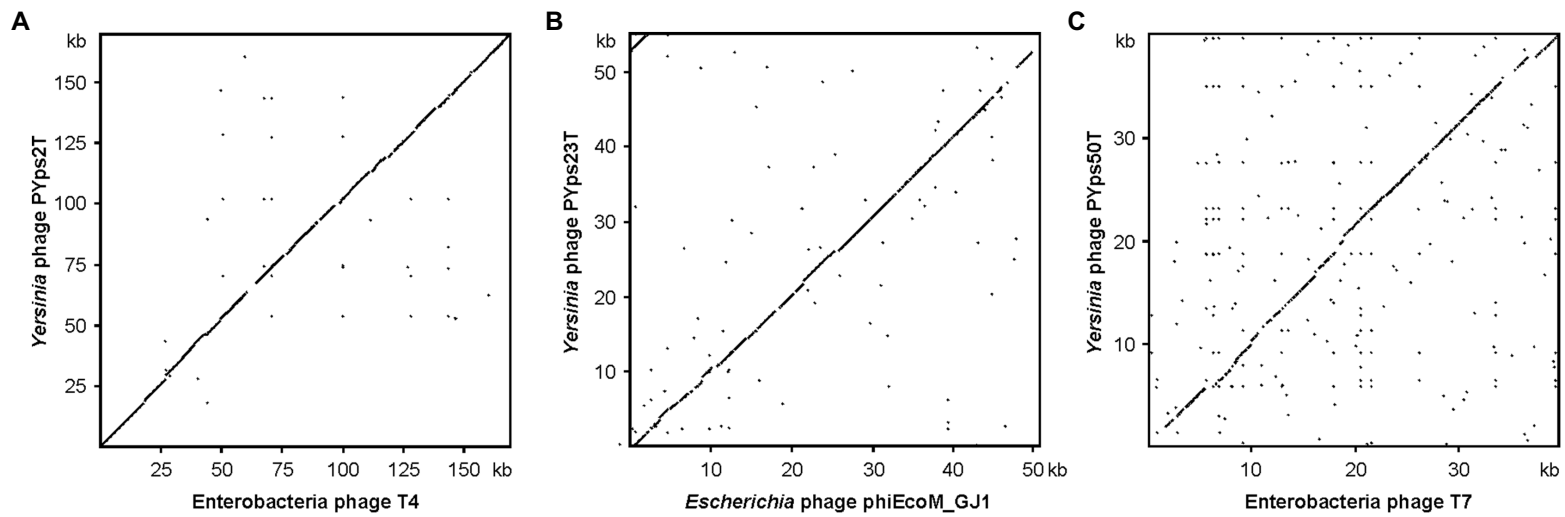
Dot plot analyses of the three *Y. pseudotuberculosis* type phages with closely related phages. **(A)** PYps2T, **(B)** PYps23T, and **(C)** PYps50T. Dot plot analyses were conducted using Accelrys DS Gene (version 2.5; Accelrys Inc.) with the following settings: minimum sequence identity: 65%; window size: 25; and hash value: 6.

**Figure 3:**
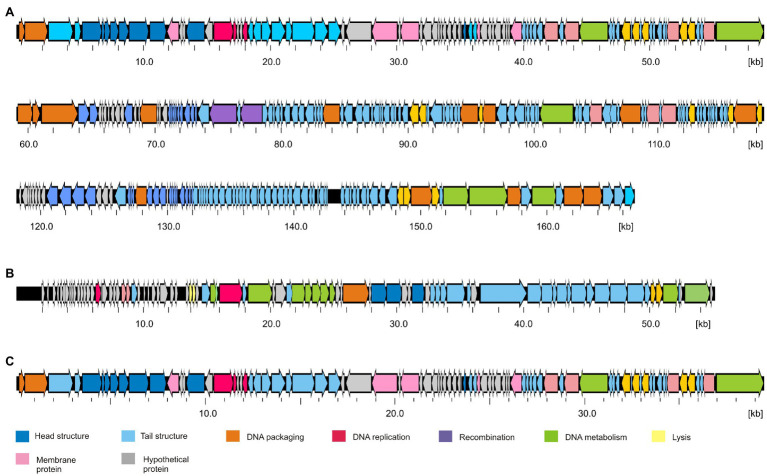
Gene maps of the *Y. pseudotuberculosis* type phages. **(A)** PYps2T, **(B)** PYps23T, and **(C)** PYps50T. Predicted functions of gene products are indicated by different colors.

**Figure 4:**
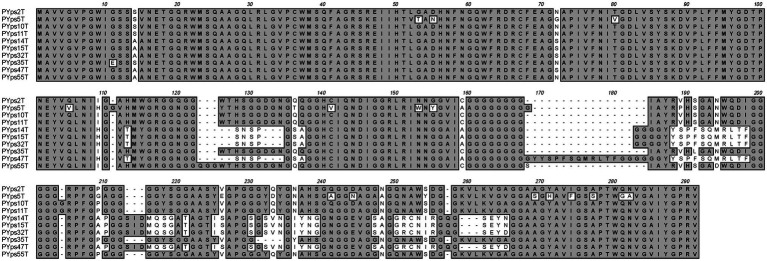
Alignment of the PYps2T group tail fiber adhesins (gp38 in T4).

The dot plot alignment of PYps2T with T4 demonstrates that both phages are very similar along their collinear genomes (97.24% identity over 88% of the genome, Figure 2A). As many T4 proteins have previously been characterized in detail, a functional prediction of 202 out of 269 PYps2T gene products could be made (Figure 3; Supplementary Table S2). Thus, a large number of genes probably involved in virion assembly, replication, or transcription were identified. In addition, 10 tRNA genes and eight homing nuclease genes were detected, which are common “molecular parasites” of T4 phage genomes that can promote their own horizontal transfer by inserting into a cognate site not containing the endonuclease gene (Sandegren and Sjoberg, 2004; Edgell et al., 2010). All PYps2T group members have a similar-sized circularly permuted genome ranging from 165,733 bp (PYps32T) to 169,493 bp (PYps10T) with a GC content of ~35.4% (Table 4). Moreover, the genome composition of the phages is very similar. Thus, the question arises why some members of this group differed so clearly in their host range lysing between 19 (PYps5T) and 106 (PYps35T) out of 128 *Y. pseudotuberculosis* strains. The gene products gp37 and gp38 of T4-related phages, which encode the long tail fiber (LTF) distal half fiber including the receptor-recognizing tip and an adhesin, respectively, have been shown to be important for phage adsorption and host specificity (Yoichi et al., 2005; Trojet et al., 2011; Storms and Sauvageau, 2014). Comparison of the large distal half fiber proteins of PYps2T group phages disclosed two subgroups, each possessing almost identical proteins (except for PYps55T that showed some amino acid exchanges), whereas phages belonging to different subgroups exhibited a sequence identity of only 80% (Supplementary Figure S1). However, there was no discernible link between the gp37 sequences and the host ranges of the phages, since the phages with the narrowest (PYps5T) and broadest (PYps35T) host range possess very similar distal half fibers. As with the gp37 products, the same subgroups could be formed by comparison of the tail fiber adhesins (gp38). Though, here, each subgroup revealed more prominent differences. The adhesins of phage PYps35T and PYps55T, e.g., contain a deletion of four and an insertion of three amino acid residues, respectively, whereas the PYps5T protein revealed a number of single amino acid exchanges (Figure 4). In the other subgroup, PYps47T differs from PYps14T, PYps15T, and PYps32T by an insertion of 15 amino acid residues. It is conceivable that these deviations are important for the host specificity of the phages.

**Table 4:** Morphological and genomic properties of the isolated *Y. pseudotuberculosis* phages.

Phage group	PYps2T										PYps23T					PYps50T	
	PYps2T	PYps5T	PYps10T	PYps11T	PYps14T	PYps15T	PYps32T	PYps35T	PYps47	PYps55T	PYps3T	PYps4T	PYps16T	PYps16N	PYps23T	PYps50T	PYps49T
Morphology (taxonomic classification)	Myovirus (Tequatrovirus)										Myovirus (Carltongylesvirus)					Podovirus (Berlinvirus)	
Genome size	169,486 bp	169,375 bp	169,493 bp	169,490 bp	166,935 bp	166,925 bp	165,733 bp	166,974 bp	165,778 bp	167,710 bp	52,854 bp^*^	52,855 bp^*^	52,855 bp^*^	52,376 bp^*^	54,869 bp	39,655 bp	39,459 bp^*^
GC content	35.4%	35.4%	35.4%	35.4%	35.4%	35.4%	35.5%	35.4%	35.5%	35.4%	43.8%^*^	43.8%^*^	43.8%^*^	44.0%^*^	43.9%	48.7%	48.8%
Genes (no.)	269	267	270	268	267	267	263	264	262	267	85	86	85	84	83	44	45
tRNA genes (no.)	10	9	10	10	10	10	10	10	10	10	0	0	0	2	1	0	0
	Arg	Arg	Arg	Arg	Arg	Arg	Arg	Arg	Arg	Arg	–	–	–	Arg	Arg	–	–
	Asn	His	Asn	Asn	Asn	Asn	Asn	Asn	Asn	Asn	–	–	–	–	–	–	–
	Tyr	Asn	Tyr	Tyr	Tyr	Tyr	Tyr	Tyr	Tyr	Tyr	–	–	–	–	–	–	–
	Met	Tyr	Met	Met	Met	Met	Met	Met	Met	Met	–	–	–	–	–	–	–
	Thr	Met	Thr	Thr	Thr	Thr	Thr	Thr	Thr	Thr	–	–	–	–	–	–	–
	Ser	Ser	Ser	Ser	Ser	Ser	Ser	Ser	Ser	Ser	–	–	–	–	–	–	–
	Pro	Pro	Pro	Pro	Pro	Pro	Pro	Pro	Pro	Pro	–	–	–	–	–	–	–
	Gly	Gly	Gly	Gly	Gly	Gly	Gly	Gly	Gly	Gly	–	–	–	–	–	–	–
	Leu	Leu	Leu	Leu	Leu	Leu	Leu	Leu	Leu	Leu	–	–	–	Leu	Leu	–	–
	Gln	–	Gln	Gln	Gln	Gln	Gln	Gln	Gln	Gln	–	–	–	–	–	–	–
Transcription terminators (no.)	84	84	84	84	82	82	91	91	91	84	20	20	20	17	22	8	8
Genome end structure	Circular permuted genome										Long terminal direct repeats					Short terminal direct repeats	
Accession number	MT828551	MT828552	MT828553	MT515751	MT526905	MT515752	MT515753	MT515754	MT515755	MT515756	MW147599	MW147600	MW147602	MW147601	MW147598	MT515757	MW147603

* The structure of the terminal ends was not determined.

### PYps23T-like Phages Belong to an Unusual Group of Myoviruses (Taxid: *Caudovirales*; *Chaseviridae*; *Carltongylesvirus*; *Escherichia* Virus ST32)

**Figure 5:**
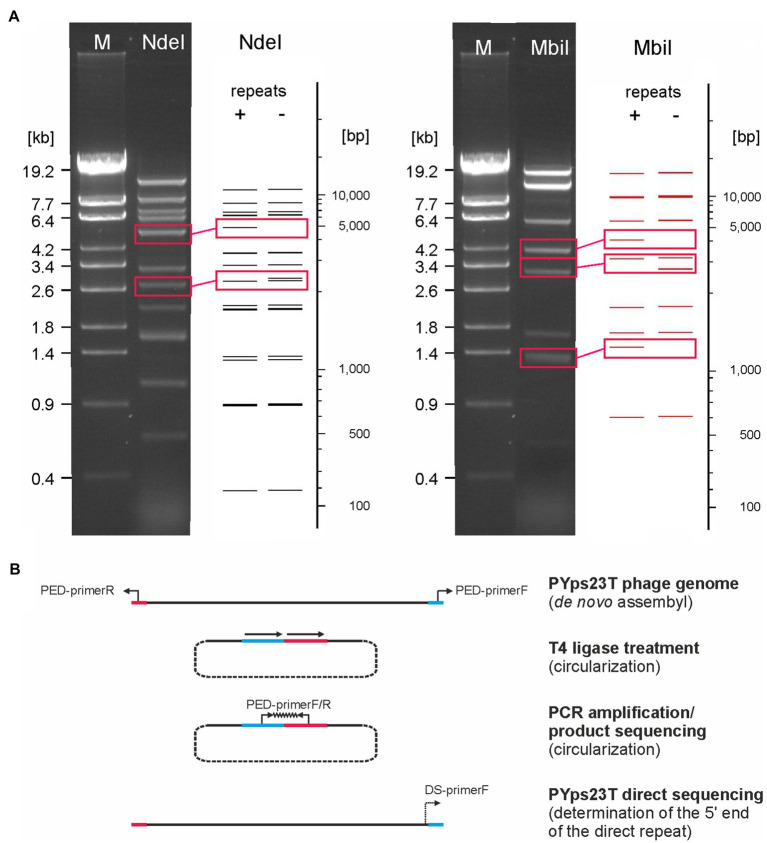
The PYps23T genome contains long terminal direct repeats. **(A)** MbiI and NdeI restriction patterns of the phage DNA obtained by agarose gel electrophoresis and by *in silico* analysis. Fragments that were present only in the gel or in the *in silico* analysis are boxed. *In silico* restriction pattern predictions of PYps23T were obtained using NEBcutter v2.0 (Vincze et al., 2003). Shown are patterns of the PYps23T genome without and with terminal repeats of 2.3 kb. M, Lambda Eco32I marker (fragment sizes are given). **(B)**. Determination of the start and end of the direct repeat. PED, PCR End Determination primer; DS, Direct Sequencing primer. See the text for details.

Members of the PYps23T group revealed strong similarities to a number of myoviruses possessing some proteins of podoviruses, e.g., *Escherichia* phage ST32, *Escherichia* phage phiEcoM-GJ1, *Pectobacterium carotovorum* phage PM1, and *Erwinia amylovora* phage vB_EamM-Y2 (Figure 2B). In type phage, PYps23T 83 predicted genes were identified and annotated, and all the predicted genes, except for one, are located on the same DNA strand. Only 43 predicted gene products could be functionally assigned, while similar to phage phiEcoM-GJ1, the predicted functions of most of the small gene products encoded by genes at the beginning of the genome are still unknown (Figure 3; Supplementary Table S3). Most PYps23T genes including a gene for a T7-RNA polymerase also exist in the same order in the other phages of this group. However, a gene encoding tRNA for arginine or leucine identified in some of these phages was only detected in two phages (PYps16N and 23T) of the PYps23T group. Interestingly, the phages PYps3T, 4T, and 16T, which were isolated 4 years earlier, lack exactly the tRNA gene, whereas adjacent DNA-sequences are almost identical to those of the other phages suggesting that the tRNA gene can be acquired or lost by horizontal gene transfer. As with most other proteins, the terminase large subunit is very similar in the PYps23T group and also in the related phages infecting other species indicating the same packaging mechanism (Liu et al., 2018). The terminases of these phages resemble proteins of phages with a T1-like headful packaging mechanism. However, while for phage phiEcoM-GJ1 a circularly permuted genome has been suggested (Jamalludeen et al., 2008), direct terminal repeats of approximately 2.6 kb have been described for the phages vB_EamM-Y2 and PM1 (Born et al., 2011; Lim et al., 2014). We therefore compared MbiI and NdeI restriction patterns of digested PYps23T DNA with patterns predicted by *in silico* analysis of the sequenced phage. For this comparison, the zero point of the PYps23T genomic sequence was determined in accordance with those of the related phages vB_EamM-Y2 and PM1. The MbiI digest showed a 4 kb fragment and an additional band of approximately 1.3 kb that were absent in the *in silico* analysis, whereas a predicted fragment of 2.8 kb was missing in the digested DNA (Figure 5A). Thus, the digested phage genome was approximately 2.5 kb larger than that determined by *in silico* analysis. This result was supported by digestion of the DNA with NdeI. Here, an additional fragment of approximately 4.8 kb appeared in the gel, while an approximately 2.3 kb fragment of a double-band predicted by *in silico* analysis was missing. To ascertain whether the additional sequence represents a direct terminal repeat, the PYps23T phage DNA was treated with T4 ligase and used for PCR analysis applying a reverse primer deduced from the predicted left end of the sequenced genome and a forward primer deduced from the predicted repeat sequence at the right end (Figure 5B). We indeed obtained a PCR product of the expected size. Sequencing of the product indicated a repeat of 2.339 bp. The repetitive sequence was confirmed by direct sequencing of phage DNA using primers deduced from the upstream region of the repetitive sequence (Figure 5B).

What phages of the PYps23T group have in common is a very similar, narrow host range. They lysed only few *Y. pseudotuberculosis* strains, but also individual strains of other species, e.g., *E. coli* (Table 2). This raises the question whether the tail fiber proteins of these phages are related to those of the *E. coli* phages ST32 and phiEcoM-GJ1. There are several proteins probably involved in the synthesis of the tail fibers. In PYps23T, Gp76 is likely a large main tail fiber protein of 1,224 amino acids, while a Gp84 may be a smaller tail fiber protein of 463 amino acids. In addition, Gp85 (tail fiber repeat protein) and Gp86 (tail fiber assembly protein) may be important for tail fiber synthesis. Though, while the main tail fiber proteins of phages belonging to the PYps23T group are 90 to 100% identical, they showed only identities of 67 and 64% to the corresponding proteins of phiEcoM-GJ1 and ST32, respectively. Moreover, the other PYps23T tail fiber-associated products are much more dissimilar to their counterparts in the *E. coli* phages with identities of 44% (Gp84) and 37% (Gp85). For Gp86, no counterpart could be detected in these phages. It is therefore unclear, why the PYps32T group can lyse *E. coli* and doubtful whether the mentioned *E. coli* phages are able to infect *Y. pseudotuberculosis* as well.

### PYps49T and PYps50T Are Phages of the Taxid: *Autographiviridae*; *Studiervirinae*; *Berlinvirus*

**Figure 6:**
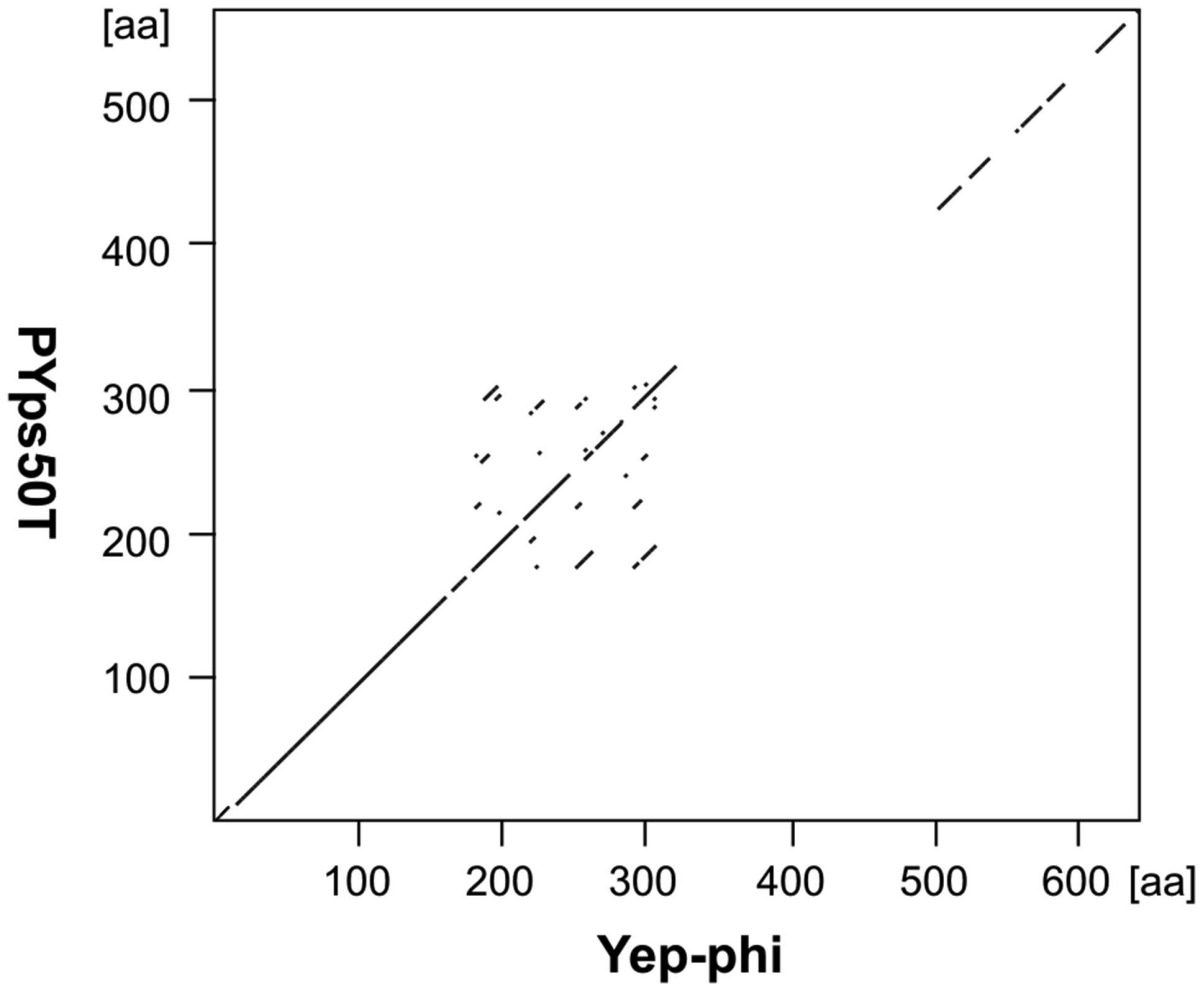
Dot plot of the tail fiber proteins of PYps50T and Yep-phi. Dot plot analyses were conducted using Accelrys DS Gene (version 2.5; Accelrys Inc.) with the following settings: minimum sequence identity: 65%; window size: 15; and hash value: 2.

These phages are closely related to phage T7 (Figure 2C). Like most other T7-like phages, the genomes of PYps49T and PYps50T contain direct terminal repeats, which have a length of 184 bp. Thirty-four of the 45 predicted PYps50T gene products could be functionally assigned. Besides a number of genes for virion assembly (head and tail proteins), some genes encoding proteins involved in DNA metabolism (e.g., for a RNA polymerase), host cell lysis, and DNA packaging were identified (Figure 3; Supplementary Table S2). The gene order is almost identical to that of other T7-related phages. Among the closest relatives of PYps50T and PYps49T are the *Y. pestis* phages Yepe2, YpP-G, Yep-phi, and Berlin, which are almost 92% identical over 85 to 86% of their covered genomes (Zhao et al., 2011). For Yep-phi, it has been reported that the phage does not infect species other than *Y. pestis* (Zhao et al., 2013). At first glance, this seems to be surprising since PYps49T and 50 T are able to lyse not only *Y. pseudotuberculosis* and *Y. pestis* but also some strains of *E. coli*, *Salmonella* and *Shigella*. A closer look at the tail fiber proteins of the phages, however, revealed that the proteins of PYps49T and 50 T are only 55% identical to those of the aforementioned *Y. pestis* phages. While the first 320 amino acids of the proteins are very similar, the next approximately 180 amino acids diverge significantly (Figure 6). Interestingly, close to the transition point from the related to the unrelated region a number of repetitive sequences are present which might have caused this break. The C-terminal part of the proteins containing the receptor binding domain is slightly similar (Zhao et al., 2013). By contrast, the PYps49T and 50T tail fiber proteins are 91% identical to that of the *Salmonella* phage BSP161. Due to the very high overall similarity to BSP161, the question arises whether also this phage is able to lyse both *Salmonella* and *Y. pseudotuberculosis*.

## Discussion

This study showed that phages able to infect *Y. pseudotuberculosis* strains are widespread in birds kept in the zoo Tierpark Berlin. From 35 to 19% of the samples collected in 2013 and 2017, respectively, phages could be isolated. All isolated phages are myoviruses or podoviruses. Though, while 10 and nine myoviruses belonging to the PYps2T and PYps23T group, respectively, were predominantly occurring, only two podoviruses were isolated, both of which in the same year. As far as we know siphoviruses (phages with long non-contractile tails) infecting *Y. pseudotuberculosis* have yet not been described, even though this group is predominant within the order *Caudovirales*. The different frequencies of positive samples may be caused by the fact that in August 2013 exclusively birds of prey and owls were studied, whereas in July 2017, also a rather large number (*n* = 18) of samples from mammals were analyzed. Thus, in that year, 28% of the samples of birds were positive for *Y. pseudotuberculosis* phages. We did not try to isolate *Y. pseudotuberculosis* from fecal samples because the animals did not show any clinical symptoms and there is no standardized protocol for such a procedure (Reinhardt et al., 2018). Nevertheless, the facts that *Y. pseudotuberculosis* phages could almost exclusively be isolated from birds and that the presence of phages is strongly associated with bacteria, in which they propagate suggests that birds are an important reservoir of hosts of those phages. In Finland, a comprehensive study on the prevalence of *Y. enterocolitica* and *Y. pseudotuberculosis* phages in pig stool was performed (Salem et al., 2015). In 90 out of 793 (11%) pig stool samples, *Yersinia* phages were detected, of which, however, only eight samples contained phages infecting *Y. pseudotuberculosis.* As *Y. pseudotuberculosis* could not be isolated in that study, this finding together with our results underlines the assumption that the enteropathogenic *Yersinia* species have different reservoirs. Though, since we did not screen the birds for *Y. pseudotuberculosis* and due to the ability of all phages to infect also non-*Yersinia* strains, it cannot be excluded that they may have replicated in other bacteria. This does not particularly concern the PYps2T group and PYps49T/PYps50T, most of them infected many *Y. pseudotuberculosis* strains. By contrast, PYps23T-like phages lysed very few *Y. pseudotuberculosis* strains but, e.g., the *E. coli* strain ATCC 35218 (Tables 1 and 2). The ability of some *Yersinia* phages to infect other *Enterobacteriaceae* has already been reported (Filippov et al., 2012; Rashid et al., 2012). However, PYps23T-related phages have yet only been described for *E. coli*, *Erwinia*, and *Pectobacterium* (Jamalludeen et al., 2008;Born et al., 2011; Lim et al., 2014), but not for *Y. pseudotuberculosis*. Therefore, it remains open, which is the natural host of PYps23T-like phages, but it is noteworthy that members of this group were isolated in 2013 and 2017 suggesting that their host is prevalent in the birds gut. Similar to closely related phages like phiEcoM-GJ1, the PYps23T group is unusual myoviruses, because they encode some podoviral proteins, particularly a T7-like single-subunit RNA polymerase, even though tRNA genes were only detected in phages isolated in 2017. Another striking difference to phiEcoM-GJ1 pertains to the proposed genome ends of the phages. Although the PYPs23T terminase large subunit is 97% identical over 99% of the protein to its counterpart in phiEcoM-GJ1, the PYps23T genome clearly contains direct terminal repeats and is not circularly permuted. Direct terminal repeats have already been reported for the phages vB_EamM-Y2 and PM1 whose terminase large subunits are also very similar to the proteins of PYps23T and phiEcoM-GJ1 (Born et al., 2011; Lim et al., 2014). We therefore speculate that all phages of this group possess terminal direct repeats at their genome ends. Compared to the PYps23T group, many more *Y. pseudotuberculosis* strains were lysed by phages belonging to the PYps2T group, isolated from 18% of the fecal samples of birds, even though the host specificity of individual members of this group diverged in part significantly (Table 1). This group is closely related to T4-like phages, particularly *Escherichia* and *Shigella* phages, but also to some *Yersinia* phages, e.g., YpsP-PST, which has an extended host range, as it lyses not only strains of *Y. pestis* and *Y. pseudotuberculosis*, but also other *Enterobacteriaceae* (Rashid et al., 2012). The wide spectrum of host specificity that we determined for members of the PYps2T group might be caused by different gp38 adhesins which are primary determinants of host range in T4-type bacteriophages (Trojet et al., 2011). Indeed, as with gp37 (LTF distal half fiber), the alignments of the gp38 adhesins of the PYps2T group revealed two subgroups. Though, there was no clear link discernable between the membership of a group and the host range. One subgroup composed of the phages PYps14T, 15T, 32T, and 47T whose gp37 and gp38 are very similar to that of YpsP-PST lysed on the average more *Y. pseudotuberculosis* strains than the other subgroup, in which, however, two members (PYps35T and PYps55T) also showed a broad-host range. It still has to be resolved, whether the insertion and deletion observed in the gp38 adhesins of PYps35T and PYps55T may affect their host specificity. In 2017, the two very similar podoviruses PYps49T and 50 T were isolated, which showed a very broad-host range and a strong lytic activity. They are related to T7-like phages, which also include some *Y. pestis* and *Y. enterocolitica* phages (Zhao et al., 2011; Salem and Skurnik, 2018). However, the tail fiber proteins of PYps49T and 50T differ significantly from those of other *Yersinia* podoviruses, which may explain why they could lyse so many strains. This property is rather unusual for podoviruses, whose host ranges are generally narrower than those of myoviruses (Wichels et al., 1998). Indeed, a cocktail containing one podovirus (PYps49T or 50T) and one myovirus (e.g., PYps35T) would be suitable to lyse 93% of the tested 128 *Y. pseudotuberculosis* strains belonging to 20 different serotypes. In addition, these two phages also lysed all tested *Y. enterocolitica* B4/O:3 strains at 37°C, whereas a significantly lower proportion (23%) of *E. coli* strains were susceptible.

## Conclusion

This study showed that zoo animals can be a promising source to isolate phages that can be used for applications to combat *Y. pseudotuberculosis.* The fact that very similar phages were isolated at an interval of 4 years indicates that they belong to the normal intestinal flora of birds. We are planning to repeat a sampling in 2022 to verify this notion and will then try to isolate *Y. pseudotuberculosis* from the samples to elucidate, whether this species may be the natural host of the phages.

## Data Availability Statement

The datasets presented in this study can be found in online repositories. The names of the repository/repositories and accession number(s) can be found in the article/Supplementary Material.

## Author Contributions

JH and SH designed the study. JH, ABa, ABi, AD, ND, CJ, NM, JJ, MS, JU, and SH performed the experiments. JH, ABa, CJ, MS, and SH analyzed the data. JH, CJ, MS, and SH prepared the tables and figures and wrote the manuscript. All authors edited the manuscript and contributed to the article and approved the submitted version.

## Funding

The work was financially supported by the Bundesinstitut für Risikobewertung (grant no. 45-002).

## Conflict of Interest

The authors declare that the research was conducted in the absence of any commercial or financial relationships that could be construed as a potential conflict of interest.

## Publisher’s Note

All claims expressed in this article are solely those of the authors and do not necessarily represent those of their affiliated organizations, or those of the publisher, the editors and the reviewers. Any product that may be evaluated in this article, or claim that may be made by its manufacturer, is not guaranteed or endorsed by the publisher.

## Footnotes

^1^ https://lpsn.dsmz.de/genus/yersinia

^2^ https://blast.ncbi.nlm.nih.gov/Blast.cgi

^3^ http://rssf.i2bc.paris-saclay.fr/toolbox/arnold/

^4^ https://cge.cbs.dtu.dk/services/CSIPhylogeny/

^5^ https://github.com/rambaut/figtree/releases/tag/v1.4.4
