# Supplementary material for: Birds Kept in the German Zoo “Tierpark Berlin” Are a Common Source for Polyvalent Yersinia pseudotuberculosis Phages
Source: Front Microbiol. 2022 Jan 3;12:634289. doi: 10.3389/fmicb.2021.634289 (PMC8762354; doi:10.3389/fmicb.2021.634289)
Supplement: Supplementary file 3 [file Table_3.docx]

Supplementary Material

# Supplementary Figure S1


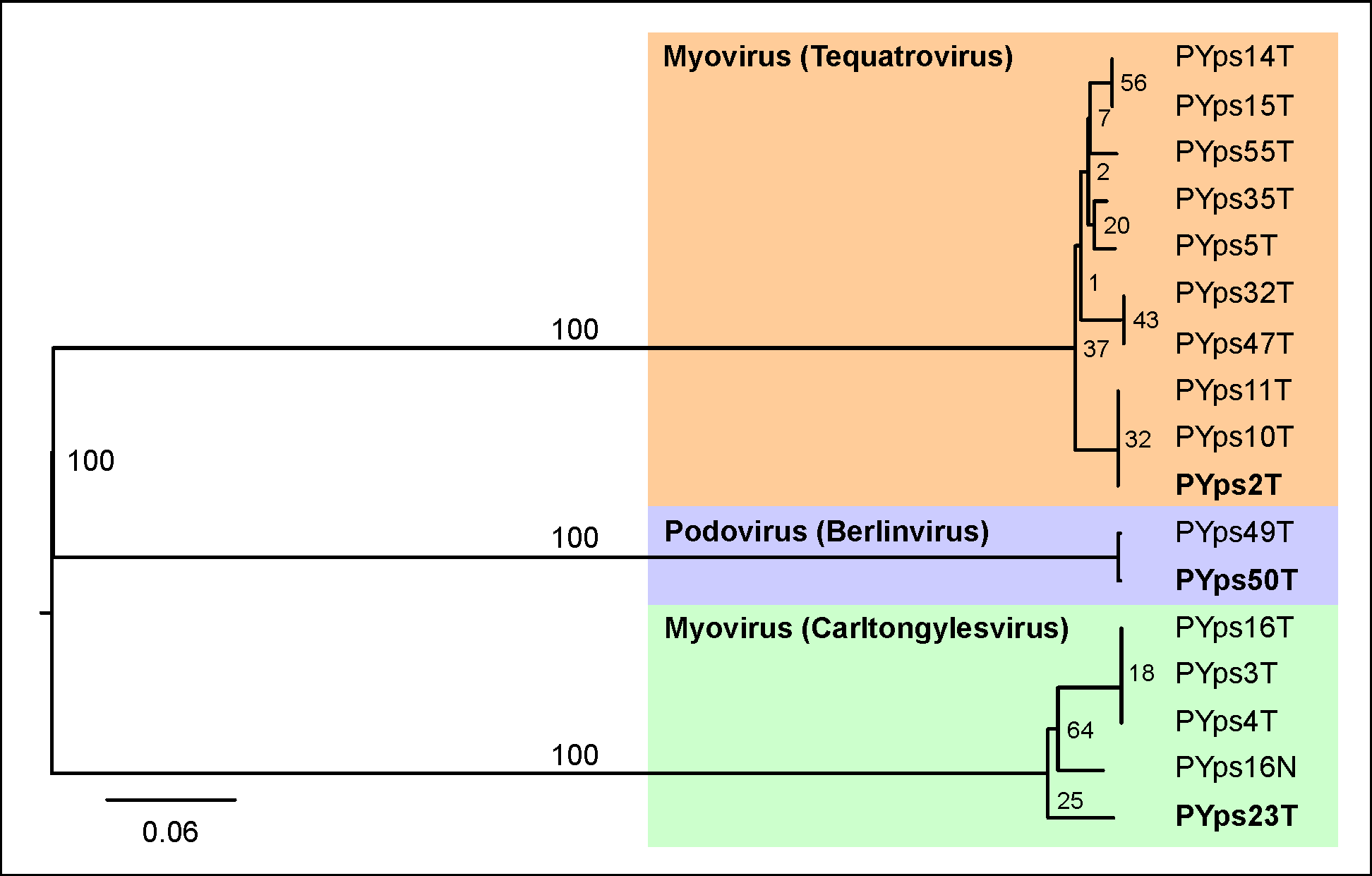


**Supplementary Figure S1.** Phylogenetic relationship of the isolated phages analyzed using the ‘VICTOR’ web service (<https://victor.dsmz.de>) as previously described (Meier-Kolthoff & Göker et al., 2017). A phylogenomic ‘Genome-BLAST Distance Phylogeny (GBDP)’ tree is shown inferred from the D0 formula and yielding an average support of 43%. Numbers given for the branches are GBDP pseudo-bootstrap support values from 100 replications. A standard for branch lengths evaluation is given. Based on an OPTSIL clustering, three clusters at genus level were assigned and colored for better visualization according to the determined virus taxons.

**Reference**

Meier-Kolthoff JP, Göker M. VICTOR: Genome-based Phylogeny and Classification of Prokaryotic Viruses. Bioinformatics 2017, 33:3396-3404.
